# Fullerene C_70_/porphyrin hybrid nanoarchitectures: single-cocrystal nanoribbons with ambipolar charge transport properties†

**DOI:** 10.1039/d2ra02669d

**Published:** 2022-07-06

**Authors:** Takatsugu Wakahara, Kahori Nagaoka, Chika Hirata, Kun'ichi Miyazawa, Kazuko Fujii, Yoshitaka Matsushita, Osamu Ito, Makito Takagi, Tomomi Shimazaki, Masanori Tachikawa, Yoshiki Wada, Shinjiro Yagyu, Yubin Liu, Yoshiyuki Nakajima, Kazuhito Tsukagoshi

**Affiliations:** Research Center for Functional Materials, National Institute for Materials Science 1-1 Namiki Tsukuba Ibaraki 305-0044 Japan Wakahara.Takatsugu@nims.go.jp; Department of Chemical Sciences and Technology, Graduate School of Chemical Sciences and Technology, Tokyo University of Science 6-3-1 Niijuku, Katsushika-ku Tokyo 125-8585 Japan; Research Network and Facility Services Division, National Institute for Materials Science 1-2-1 Sengen Tsukuba Ibaraki 305-0047 Japan; Quantum Chemistry Division, Graduate School of NanoBioScience, Yokohama City University 22-2 Seto, Kanazawa-ku Yokohama Kanagawa 236-0027 Japan tachi@yokohama-cu.ac.jp; RIKEN KEIKI Co., Ltd 2-7-6, Azusawa Itabashi-ku Tokyo 174-8744 Japan; International Center for Materials Nanoarchitectonics (WPI-MANA), National Institute for Materials Science 1-1 Namiki Tsukuba Ibaraki 305-0044 Japan Tsukagoshi.Kazuhito@nims.go.jp

## Abstract

In recent years, supramolecular cocrystals containing organic donors and acceptors have been explored as active components in organic field-effect transistors (FETs). Herein, we report the synthesis of novel single-cocrystal nanoribbons with ambipolar charge transport characteristics from C_70_ and 5,10,15,20-tetrakis(3,5-dimethoxyphenyl)porphyrin (3,5-TPP) in a 3 : 2 ratio. The C_70_/3,5-TPP nanoribbons exhibited a new strong absorption band in the near-infrared region, indicating the presence of charge-transfer interactions between C_70_ and 3,5-TPP in the cocrystals. We elucidated the mechanism of the charge-transport properties of the nanoribbons using photoemission yield spectroscopy in air and theoretical calculations. A strong interaction between porphyrins in the one-dimensional porphyrin chains formed in C_70_/3,5-TPP nanoribbons, which was confirmed by single-crystal X-ray diffraction, plays a crucial role in their hole transport properties.

## Abstract 2

The one-dimensional porphyrin chains in the cocrystal play a very important role in the hole transport properties of C_70_/porphyrin nanoribbons.

## Graphical abstract

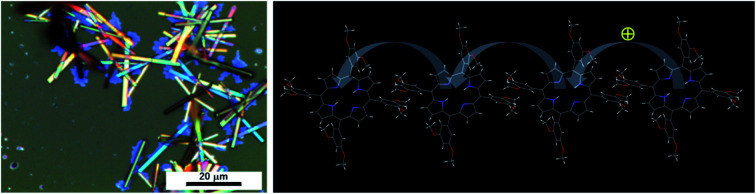

## Introduction

Functional supramolecular nanoarchitectures have tremendous potential as building blocks for bottom-up organic nanoscale electronic devices^1–3^ such as transistors.^4^ Recently, several studies have been reported on transistors employing supramolecular nanoarchitectures, such as nanowhiskers, nanotubes, nanorods, nanowires, and nanosheets.^1,5,6^ A distinct characteristic of almost all these reported transistors is that they transport only a single carrier type, *i.e.*, either holes (p-type) or electrons (n-type). Therefore, simultaneous or selectable transport of electrons and/or holes (ambipolar charge transport) remains a highly desirable device characteristic to be achieved, because this characteristic will be able to facilitate the design of better-performing electronic circuits^7^ as well as the demonstration of bifunctional organic devices, such as light-emitting^8^ and light-sensing transistors.^9^ In 2012, we reported novel ambipolar FETs based on C_60_/5,10,15,20-tetrakis(4-methoxyphenyl)porphyrinato cobalt(ii) (C_60_/CoTMPP) cocrystals.^10^ Zhu *et al.* also reported ambipolar FETs using sulfur-bridged annulene-TCNQ cocrystals.^11^ Following these earlier studies, various FETs based on donor–acceptor (D–A) cocrystals have been reported.^12–14^ There are several reports on ambipolar FETs based on novel organic semiconductors in the literature;^15,16^ however, the preparation of these semiconductors requires complex organic syntheses. In contrast, the co-crystallization process can be conducted by simple mixing of two (or more) molecules and does not involve any complex synthesis protocols. Notably, not all D–A cocrystal-based FETs exhibit ambipolar transport properties, some exhibit the unipolar ones, such as n-type^17–20^ or p-type.^21^ Therefore, further research is necessary to understand these characteristics in detail.

Herein, we report the preparation of cocrystal nanoribbons from C_70_ and 5,10,15,20-tetrakis(3,5-dimethoxyphenyl)porphyrin (3,5-TPP) as a pair of electron-acceptor and electron-donor molecules by a simple solution mixing method. These C_70_/3,5-TPP nanoribbons were used to fabricate bottom-gate/bottom-contact FETs. Despite using a non-optimized device architecture, these FETs exhibited ambipolar transport characteristics. This result is in sharp contrast with our earlier report on C_60_ analogue (C_60_/3,5-TPP cocrystals) that showed only n-type behavior.^17^ We found that the one-dimensional porphyrin chains formed by the strong interaction of porphyrins inside the nanoribbons play a very important role in the hole transport properties of the C_70_/3,5-TPP nanoribbons.

## Experimental section

### Preparation and characterization of C_70_/3,5-TPP nanoribbons

C_70_/3,5-TPP nanoribbons were prepared by mixing two solutions containing C_70_ and 3,5-TPP. Specifically, 3 ml of C_70_-saturated toluene solution was placed in a 9 ml glass bottle and 1 mg of 3,5-TPP in 1 ml of toluene was added to this solution. The mixed solution was then vigorously shaken and stored at 10 °C for 24 h to grow the C_70_/3,5-TPP nanoribbons. C_70_/3,5-TPP nanoribbons were collected by filtering the solution and washing with iso-propyl alcohol (IPA). These nanoribbons were stable in dark conditions at room temperature for long periods.

The structure and morphology of the obtained nanoribbons were analyzed by scanning electron microscopy (SEM; JEOL JSM-6700F, 5 kV), atomic force microscopy (AFM, JEOL, JSPM-5200), a micro-Raman system (Photon Design) equipped with a semiconductor laser with a wavelength of 532 nm, and a single crystal diffractometer (Rigaku Saturn CCD) using Mo Kα X-ray radiation at 113 K. The diffuse reflectance spectra of the nanoribbons were recorded using a UV-Vis-NIR spectroscope (JASCO V-570) equipped with an integrating sphere. Photoemission yield spectroscopy in air (PYSA) was performed using a RIKEN KEIKI AC-3 photoelectron spectrometer equipped with a monochromatic D2 lamp.

The time-resolved luminescence (TRPL) spectra of materials were measured by excitation with a pulsed laser (100 fs pulse width, 1 kHz repetition frequency) generated by an optical parametric amplifier (Spectra Physics TOPAS Prime). The excitation wavelength for TRPL spectroscopy analysis was 520 nm.

Transistors were fabricated using a previously reported method.^17^ The electrical transport properties were measured inside a glove box using an Agilent B2902A and Agilent E5272A.

For the first-principles quantum chemical calculations, the Gaussian16 (Revision A.03) program package^22^ was employed together with our Python-based quantum chemical tool.^23^ The electronic structures were obtained by using the density functional theory (DFT) at the B3LYP/6-31G(d) level. The molecular orbitals were depicted by using Chemcraft.^24^

## Results and discussion

**Fig. 1:**
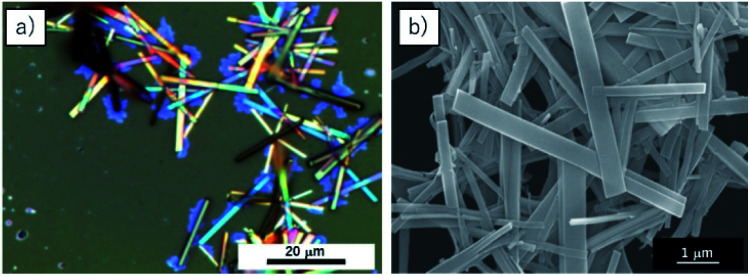
(a) Optical microscopy image and (b) SEM image of C_70_/3,5-TPP nanoribbons.

**Fig. 2:**
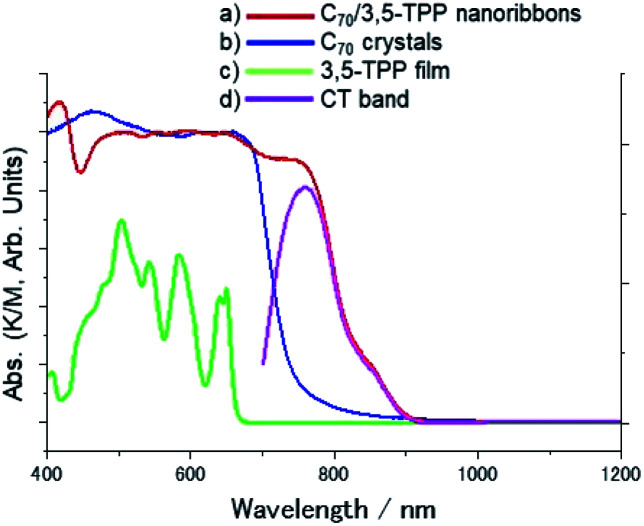
Diffuse reflectance spectra in Vis and near-infrared regions (K/M: Kubelka–Munk function, which is proportional to absorbance); (a) C_70_/3,5-TPP nanoribbons, (b) C_70_ crystals, (c) 3,5-TPP film, (d) subtraction of normalized absorption spectra at 610 nm as shown in (a) and (b).

The nanoribbons are classified as cocrystals of C_70_ and 3,5-TPP as they are single-phase crystalline materials, composed of two different molecules. Fig. 1 shows optical microscopy and SEM images of the C_70_/3,5-TPP nanoribbons. Both the images show parallelogram morphology of the nanoribbons. The length and width of the nanoribbons were found to be 15–20 μm and 0.2–0.5 μm, respectively (Fig. S1†). The thickness of the nanoribbons, as determined by SEM and AFM measurements, was in the range of 20–200 nm (Fig. S2†). Fig. 2 shows the diffuse reflectance spectra of the C_70_/3,5-TPP nanoribbons, C_70_ crystals, and 3,5-TPP film. It is observed that the C_70_ crystals prepared using the liquid–liquid interface precipitation method^17^ showed a broad absorption in the 700–400 nm region, whereas the 3,5-TPP film exhibited strong and weak absorption at approximately 400 nm and 500–600 nm, respectively. The C_70_/3,5-TPP cocrystals exhibited a new absorption in the longer wavelength region (up to 900 nm). Subtraction of the normalized absorption spectrum of C_70_ from that of the C_70_/3,5-TPP cocrystals clearly showed the extra absorption in the range of 900–700 nm (with an absorption maximum at 760 nm), which was referred to as the charge-transfer (CT) absorption band of the C_70_/3,5-TPP nanoribbons.

**Fig. 3:**
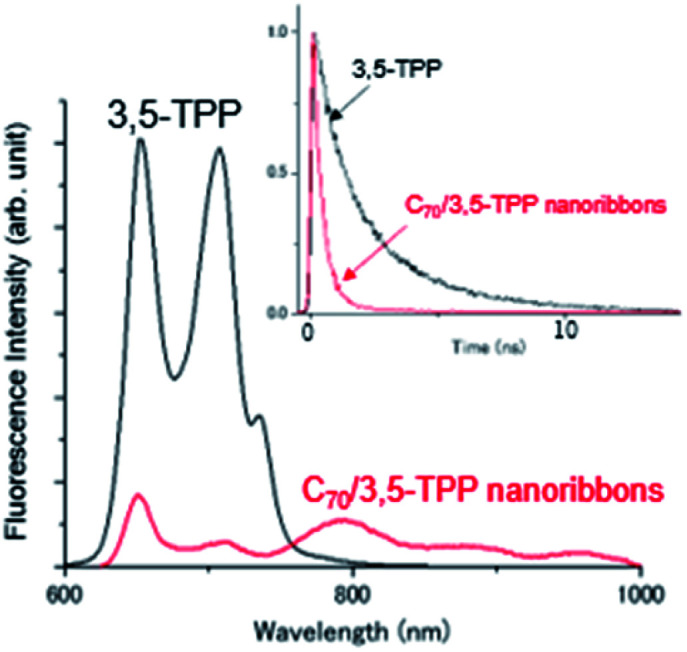
Steady-state fluorescence spectra of 3,5-TPP crystals (black) and C_70_/3,5-TPP nanoribbons (red); *λ*_ex_: 520 nm. Inset: Time profiles of fluorescence intensity [*λ*_ex_: 520 nm, *λ*_em_: 690–730 nm for 3,5-TPP crystals (black)^17^ and *λ*_em_: 780–820 nm for C_70_/3,5-TPP nanoribbons (red)].

To understand the CT characteristics under light illumination, we measured the photoluminescence (PL) spectra of the C_70_/3,5-TPP cocrystals (Fig. 3). The CT fluorescence band was observed in the range of 750–1000 nm with a peak at 800 nm, as a mirror image of the CT absorption band at 760 nm. These CT emission peaks are apparently in longer wavelength regions than the emission of C_70_-crystals.^25^ A similar CT band was reported for C_60_/3,5-TPPcocrystals.^17^ Based on the appearance of the CT absorption band, we conclude that C_70_ molecules interact with the nearest 3,5-TPP molecules. These interactions in the ground state lead to the formation of the C_70_/3,5-TPP cocrystals with alternative arrangement of C_70_ and 3,5-TPP.

The fluorescence lifetime of the C_70_/3,5-TPP cocrystals was evaluated from the decay–time profile measured using time-resolved luminescence (TRPL) spectroscopy (inset of Fig. 3). Based on the luminescence decay at 770–810 nm, the lifetime of the CT luminescence was measured as approximately 0.5 ns, which is shorter than those of the 3,5-TPP crystal (approximately 2.5 ns) and C_60_/3,5-TPP cocrystals (approximately 1.0 ns), which is calculated using the previously reported data.^17^ The observed CT luminescence lifetime suggests that the short CT-excited lifetime is due to rapid shift in electron distribution from the donor to the acceptor part in the excited CT state.

**Fig. 4:**
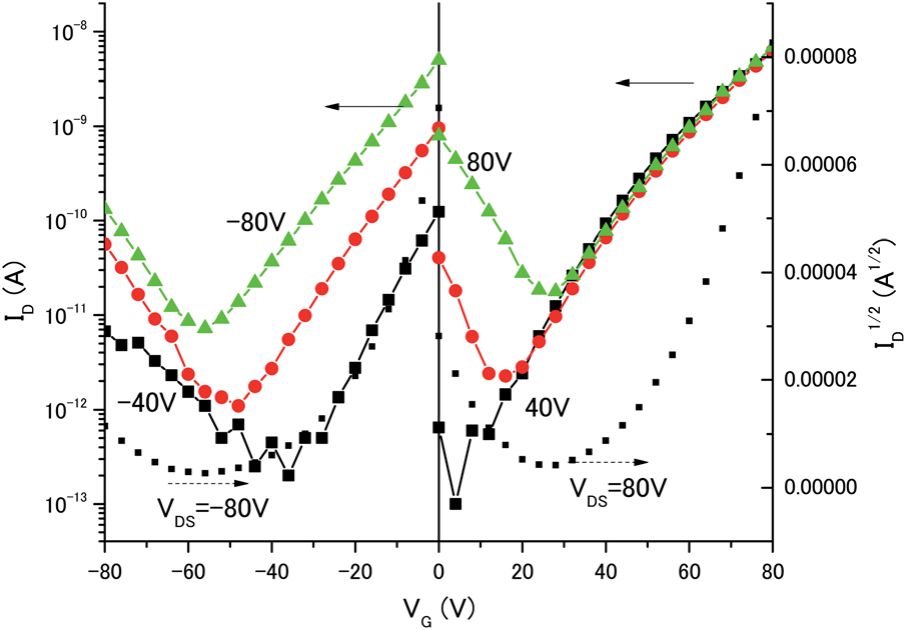
Transfer (*I*_D_–*V*_G_) characteristics of C_70_/3,5-TPP nanoribbons recorded in dark conditions. The solid curve displays drain current (*I*_D_) as a function of gate voltage (*V*_G_), and the dotted curve shows the square root of *I*_D_ (right vertical axis).

To investigate the charge transport properties of the C_70_/3,5-TPP nanoribbons, we fabricated bottom-gate, bottom-contact FETs by drop-casting an IPA solution containing C_70_/3,5-TPP nanoribbons onto a substrate. The substrate used was pre-patterned with gold source-drain electrodes with a channel length and width of 2–10 μm and 10 000 μm, respectively, and SiO_2_ (300 nm thick) as the gate dielectric. The Au electrodes were treated with self-assembled monolayers of undecanethiol and the SiO_2_ dielectric interface was rendered hydrophobic by treatment with hexamethyldisilazane. This fabrication method, as developed by Samori *et al.*,^26^ is simple and convenient for the qualitative charge-transport characterization of the micrometer-sized single-crystals. Fig. 4 shows the dependence of the drain current (*I*_D_) on the gate voltage (*V*_G_) of the C_70_/3,5-TPP nanoribbon FET. The measurements were performed under N_2_ atmosphere at room temperature under dark conditions after annealing at 353 K. (By annealing at 353 K, we observed the increase of channel current of the devices. The XRD pattern measured after annealing showed that the crystal structure is unchanged even after annealing (Fig. S3†).) The transfer characteristics of the C_70_/3,5-TPP nanoribbon-based FETs showed a V-shape in which the two arms indicated electron transport (n-type) and hole transport (p-type), respectively. These results are in sharp contrast with previously reported measurements on C_60_ nanowhisker^5^- and C_60_/3,5-TPP cocrystal^17^-based FETs that showed only n-type behavior. The electron and hole mobilities calculated from the data in Fig. 4 were relatively low, in the order of 8.2 × 10^−5^ cm^2^ V^−1^ s^−1^ and 3.1 × 10^−6^ cm^2^ V^−1^ s^−1^, respectively. However, we believe that further device optimization can lead to higher carrier mobilities.

There are many studies in the literature on D–A cocrystal-based FETs reporting n-type, p-type, or ambipolar characteristics;^17–21^ however, their characteristics and charge transport mechanisms have not yet been fully understood. In order to understand the mechanism of the ambipolar charge transport properties, a comparison of C_70_/3,5-TPP cocrystals observed in the present study with our earlier report on C_60_/3,5-TPP cocrystals^17^ that showed only n-type behavior may be useful.

At first, to realize their electronic structure, we carried out PYSA measurements of C_70_/3,5-TPP and C_60_/3,5-TPP cocrystals, 3,5-TPP, and C_70_ powder. PYSA is a powerful tool for understanding the electronic and electrical properties of molecular semiconductors.^27–29^ Recently, we succeeded in evaluating the electronic structure of C_60_/Fc nanosheets with high sensitivities using PYSA.^30^

**Fig. 5:**
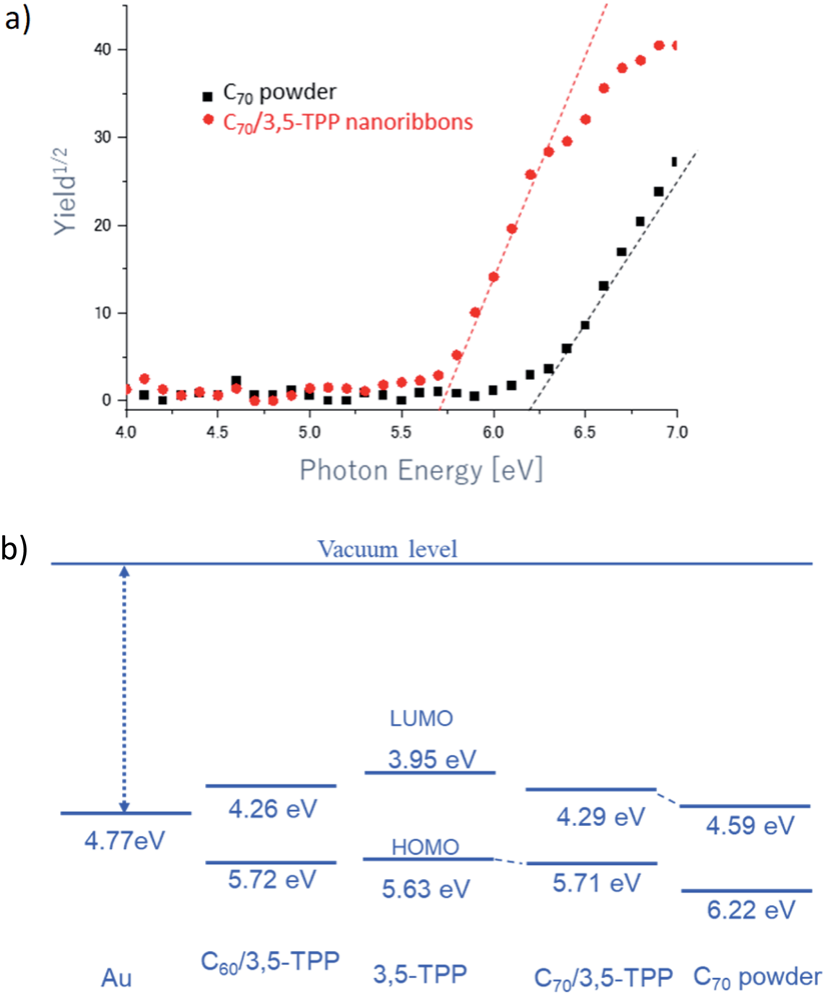
(a) PYSA spectra and (b) energy level diagrams of C_60_/3,5-TPP cocrystals, 3,5-TPP, C_70_/3,5-TPP nanoribbons, and C_70_ powder. For semi-conductor, the HOMO and the LUMO correspond to the top of the valence band and bottom of the conduction band, respectively.

The ionization energy (*I*_s_) of the C_70_/3,5-TPP nanoribbons was determined to be 5.71 eV, which is 0.51 eV smaller than that of the C_70_ powder, similar to that of 3,5-TPP. Fig. 5b shows the energy level diagrams of the C_70_/3,5-TPP nanoribbons, C_60_/3,5-TPP cocrystals, 3,5-TPP, and C_70_, estimated using the measured values of *I*_s_ and the energy gap (*E*_g_). The *E*_g_ values were obtained from the Vis-NIR absorption spectra measured in the present study for the C_70_/3,5-TPP nanoribbons and the previous study for C_60_/3,5-TPP cocrystals.^17^ The energy level diagrams demonstrate that the cocrystallization of C_70_ with 3,5-TPP enhanced the p-type FET characteristics because of the decreased hole injection barrier between the gold electrode and the highest occupied molecular orbital (HOMO) of the C_70_/3,5-TPP nanoribbons. Similarly, the *I*_s_ of the C_60_/3,5-TPP cocrystals was determined to be 5.72 eV (Fig. S4†), and the calculated energy level of the lowest unoccupied molecular orbital (LUMO) of the C_60_/3,5-TPP cocrystals is located close to that of C_70_/3,5-TPP nanoribbons. This indicates that the decrease in the hole injection barrier also takes place in the C_60_/3,5-TPP cocrystal-FET. Therefore the difference between the charge transport properties of ambipolar C_70_/3,5-TPP nanoribbons and unipolar (n-type) C_60_/3,5-TPP cocrystals cannot be explained only by the decrease in the hole injection barrier. Consequently, some other factors such as structural difference must be considered.

**Fig. 6:**
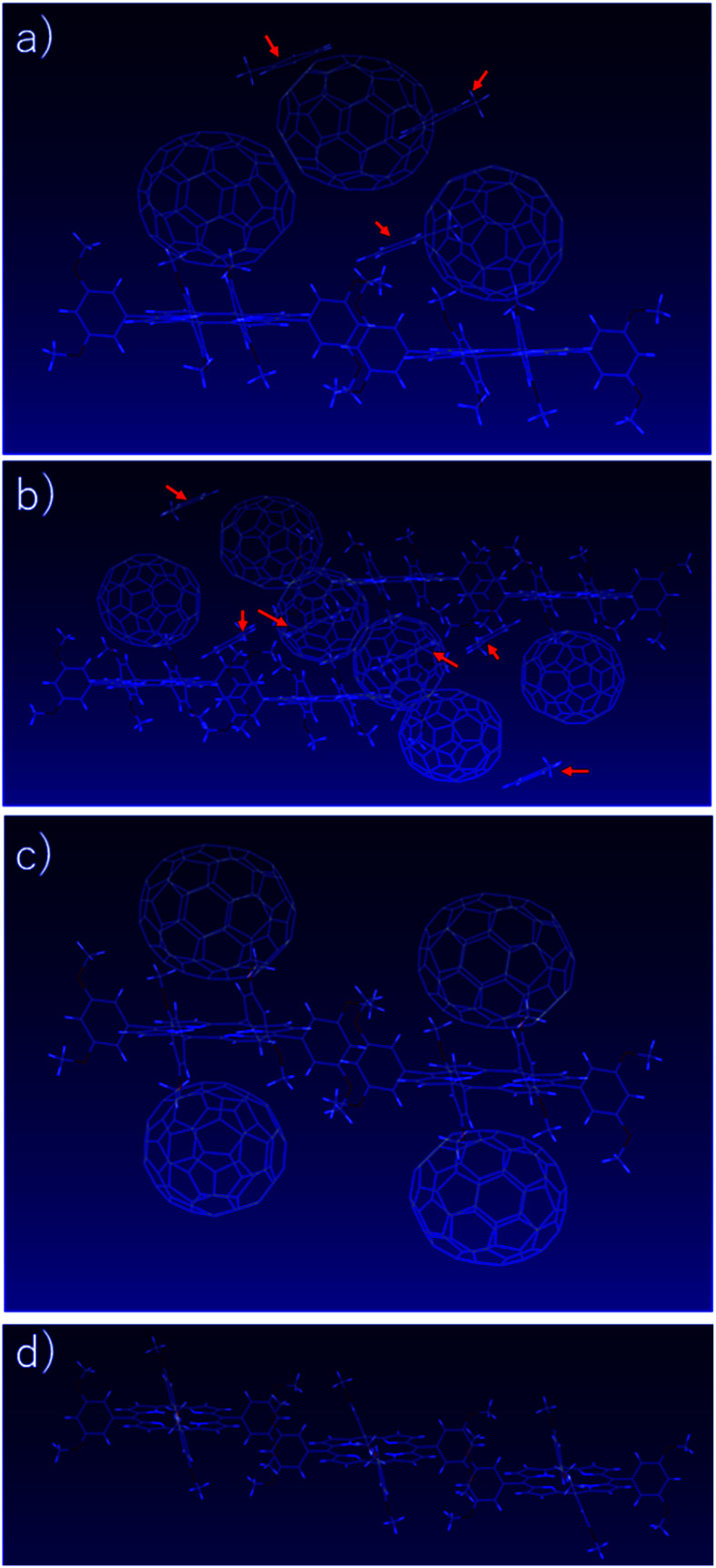
Crystal structure of a C_70_/3,5-TPP nanoribbons, (a) [(3,5-TPP)_2_·(C_70_)_3_·(toluene)_3_] unit structure (b) [(3,5-TPP)_2_·(C_70_)_3_·(toluene)_3_]_2_ unit structure (c) the [C_70_/(3,5-TPP)/C_70_]_2_ unit structure, and (d) one-dimensional porphyrin chains unit structure. Red arrows indicate the toluene molecules in the crystals.

To determine the relationship between the charge transport properties and the molecular arrangement in the C_70_/3,5-TPP nanoribbon, we determined the crystal structure of the C_70_/3,5-TPP nanoribbon by single-crystal XRD at 113 K (Fig. 6a). Crystal structure of the C_70_/3,5-TPP nanoribbon belongs to triclinic *P̄*1 space group with the unit cell: *a* = 13.8828(3) Å, *b* = 20.9103(5) Å, *c* = 30.2321(7) Å, *α* = 84.678(1)°, *β* = 86.015(1)°, and *γ* = 84.537(1)° (CCDC-2161616) (Fig. 6). The cocrystal was formulated as [(3,5-TPP)_2_·(C_70_)_3_·(toluene)_3_] and contained a [C_70_/(3,5-TPP)/C_70_]_2_ unit, in which four C_70_ molecules assembled above and below the two 3,5-TPP molecular planes (Fig. 6c). The C_70_ molecules were found to be arranged in zigzag chains that render effective electron transport from one C_70_ to a nearby C_70_. This type of [fullerene/(3,5-TPP)/fullerene)] substructure was also observed in the C_60_/3,5-TPP cocrystal.^17^ One of the biggest structural differences between the C_70_/3,5-TPP nanoribbons and C_60_/3,5-TPP cocrystals is the distance between the nearest 3,5-TPP molecules in the crystal structure. In the C_70_/3,5-TPP nanoribbons, the 3,5-TPPs were found to be located very close to each other and interacted with the 3,5-dimethoxyphenyl group, resulting in the formation of one-dimensional porphyrin chains (Fig. 6d). The center-to-center distance between the two 3,5-TPP was measured to be approximately 15 Å. Based on the observed results, the most probable mode for electric conduction stems from these porphyrin chains which act as hole-transport pathways in the C_70_/3,5-TPP nanoribbons by the hopping conduction. One-dimensional porphyrin chains are also formed in C_60_/3,5-TPP cocrystals. However, the distance between the two 3,5-TPP is approximately 21 Å, because two toluene molecules exist between two 3,5-TPP molecules, which reduces the interaction of the 3,5-TPPs (Fig. S5†). As a result, the C_60_/3,5-TPP co-crystal exhibited only n-type behavior. Whereas, in the C_70_/3,5-TPP nanoribbons, toluene molecules are located above the 3,5-TPP molecules (Fig. 6a and b and S6†). These geometrical differences may be related to the fact that ambipolar transport characteristics were observed only for the C_70_/3,5-TPP nanoribbons, but not for the C_60_/3,5-TPP cocrystals.

**Fig. 7:**
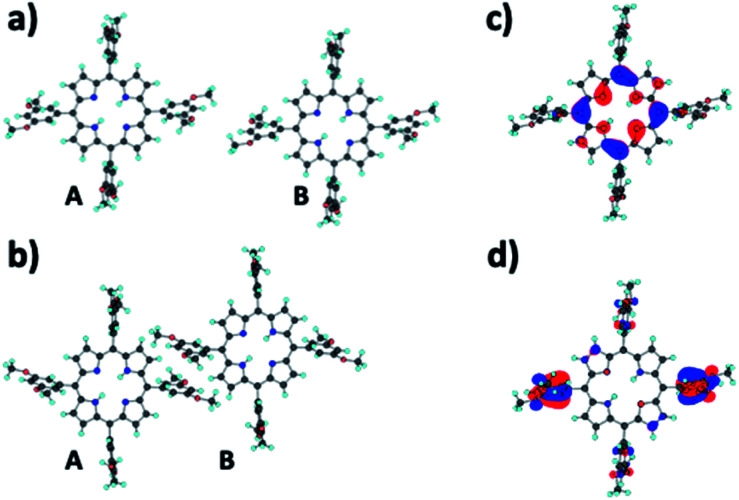
TPPs structures extracted (a) C_60_/3,5-TPP and (b) C_70_/3,5-TPP cocrystals. The distance between porphyrins in C_70_/3,5-TPP is shorter than that in C_60_/3,5-TPP. The molecular orbital of (c) HOMO and (d) HOMO-3, which are localized at the center and side chains of TPP, respectively. The interaction between side chains of 3,5-TPPs gives a larger coupling constant.

For better understanding the hole transport mechanism of cocrystals, we estimated the coupling constant between 3,5-TPPs, based on the DFT method. Fig. 7a and b show inter-molecular structures extracted from C_60_- and C_70_-based cocrystals with 3,5-TPP, respectively, where TPPs of the C_70_-based cocrystal are more densely packed, and the distance between side chains of porphyrin derivatives is shorter, compared with those of C_60_/3,5-TPP. The coupling constant rapidly decreases with inter-molecular distances, and therefore hole can more easily transport in the C_70_/3,5-TPP nanoribbons. The first-principles quantum chemical method gives the coupling constant of 4.1 × 10^−4^ and 5.3 × 10^−2^ eV between TPPs of C_60_- and C_70_-based cocrystals, respectively. Here, the coupling constant is estimated from 〈*ϕ*_A_|*H*_AB_|*ϕ*_B_〉, where *ϕ*_A(B)_ is the molecular orbital of each TPP molecule, and *H*_AB_ is the Hamiltonian of the whole molecular system. We note that there are several techniques to estimate coupling constants. For example, Krysko *et al.* employed the multireference quantum chemistry method.^31^ The generalized Mulliken–Hush method was adopted to calculate electronic couplings for hole transfers in DNA.^32^ In the coupling constant calculations for the hopping process, the localization of hole may be a key factor. This paper assumes that a hole is localized on each 3,5-TPP site. Fig. 7c and d show that the molecular orbitals of HOMO and HOMO-3 are localized at the center and side-chains of 3,5-TPP, respectively. The coupling constant has a larger value between molecular orbitals localized on side chain, such as seen in Fig. 7d. However, the coupling constant between HOMOs is small (8.8 × 10^−4^ eV) even in the C_70_/3,5-TPP case. We also note that the coupling constant related to HOMO-2 becomes relatively large because of localized orbitals on side chains. The details on molecular orbitals and coupling constants are summarized in the ESI (Fig. S7 and Table S1†). The molecular design on the side chain may be essential to control fast hole transports. Conversely, the large distance between side chains of C_60_/3,5-TPPs disturbs efficient hole transfers, and results in the n-type behavior of the cocrystal. Thus, the hole conductivity of cocrystal materials is also controlled by higher-order structures such as nanoribbons.

## Conclusions

We successfully constructed supramolecular nanoarchitectures (nanoribbons) comprising C_70_ and 3,5-TPP in a 3 : 2 ratio using a simple solution mixing method. The C_70_/3,5-TPP nanoribbons exhibited ambipolar transport characteristics with nearly balanced hole/electron mobilities. This result is in sharp contrast with our earlier report on C_60_/3,5-TPP cocrystals, which showed only n-type behavior. Single-crystal X-ray diffraction showed that one-dimensional porphyrin chains with zigzag C_70_ arrangement are formed in C_70_/3,5-TPP cocrystals. Our theoretical calculations show the one-dimensional porphyrin chains formed by the strong interactions through the side chains of porphyrins play a very important role in the hole transport properties of C_70_/3,5-TPP nanoribbons, in addition to the low electron injection barrier. Meanwhile, in the case of C_60_/3,5-TPP cocrystals, the interaction between porphyrins is reduced owing to the presence of two toluene molecules between porphyrins, which hinder hole transport from one porphyrin to a nearby porphyrin in the C_60_/3,5-TPP cocrystals. These observations are also supported by the theoretical calculations.

In recent years, numerous organic semiconductors with narrow bandgaps have been synthesized to fabricate ambipolar FETs. In the present study, we developed a distinctive method to prepare ambipolar materials without synthesizing new narrow-bandgap organic semiconductors. The successful preparation of nanoarchitectures with unique electronic characteristics by a simple co-crystallization method can be considered as an important stepping stone toward the fabrication of novel nanoscale devices.

## Author contributions

K. N. and C. H. fabricated and characterized the devices. K. i. N., K. F., and O. I. contributed to the interpretation of the experimental results. Y. M. performed single-crystal XRD analysis. Y. W. measured TRPL spectra. M. T. and T. S. performed theoretical calculations. S. Y., Y. L., and Y. N. measured the PYSA. M. T., K. T., and T. W. conducted the experiments and prepared the manuscript. All authors contributed equally to write and approve the final version of the manuscript.

## Conflicts of interest

There are no conflicts to declare.

## Supplementary Material

RA-012-D2RA02669D-s001

RA-012-D2RA02669D-s002
