# Supplementary material for: Fullerene C70/porphyrin hybrid nanoarchitectures: single-cocrystal nanoribbons with ambipolar charge transport properties
Source: RSC Adv. 2022 Jul 6;12(30):19548–53. doi: 10.1039/d2ra02669d (PMC9258400; doi:10.1039/d2ra02669d)
Supplement: RA-012-D2RA02669D-s001 [file RA-012-D2RA02669D-s001.pdf]

Electronic Supplementary Material (ESI) for RSC Advances.
This journal is © The Royal Society of Chemistry 2022

Supporting Information

Fullerene C_{70}/Porphyrin Hybrid Nanoarchitectures: Single-Cocrystal Nanoribbons with Ambipolar Charge Transport Properties

Takatsugu Wakahara,*[a] Kahori Nagaoka,[a] Chika Hirata,[a] Kun'ichi Miyazawa,[b] Kazuko Fujii,[a] Yoshitaka Matsushita,[c] Osamu Ito,[a] Makito Takagi,[d] Tomomi Shimazaki,[d] Masanori Tachikawa,*[d] Yoshiki Wada,[a] Shinjiro Yagyu,[a] Yubin Liu,[e] Yoshiyuki Nakajima,[e] and Kazuhito Tsukagoshi*[f]

[a] *Research Center for Functional Materials, National Institute for Materials Science 1-1 Namiki, Tsukuba, Ibaraki 305-0044, Japan.*

E-mail: Wakahara.Takatsugu@nims.go.jp

[b] *Department of Chemical Sciences and Technology, Graduate School of Chemical Sciences and Technology, Tokyo University of Science, 12-1-203, Ichigaya Funakawara-machi, Shinjuku-ku, Tokyo 162-0826, Japan*

[c] *Research Network and Facility Services Division, National Institute for Materials Science, 1-2-1 Sengen, Tsukuba, Ibaraki 305-0047, Japan*

[d] *Quantum Chemistry Division, Graduate School of NanoBioScience, Yokohama City University, 22-2 Seto, Kanazawa-ku, Yokohama, Kanagawa 236-0027, Japan*

E-mail: tachi@yokohama-cu.ac.jp

[e] *RIKEN KEIKI Co.,Ltd., 2-7-6, Azusawa, Itabashi-ku, Tokyo, 174-8744, JAPAN*

[f] *International Center for Materials Nanoarchitectonics (WPI-MANA),National Institute for Materials Science, 1-1 Namiki, Tsukuba, Ibaraki 305-0044, Japan*

E-mail: Tsukagoshi.Kazuhito@nims.go.jp

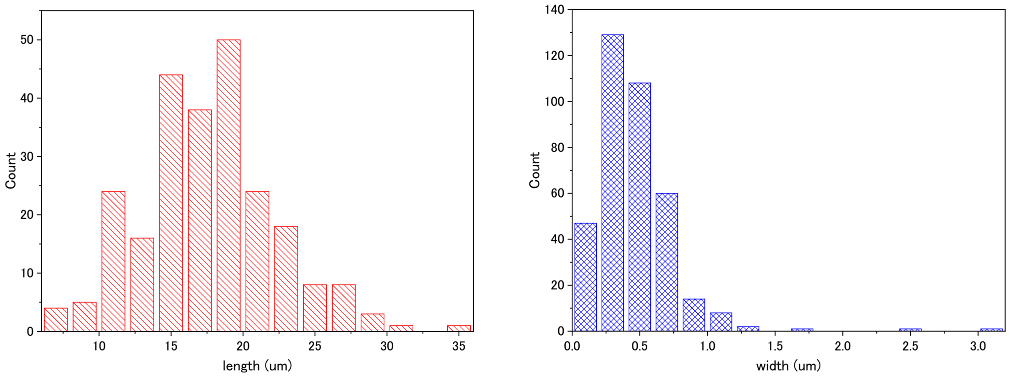

Figure S1. The size distribution histograms of C_{70}/3,5-TPP nanoribbons.

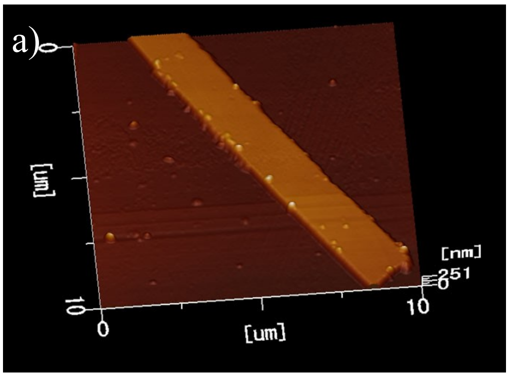

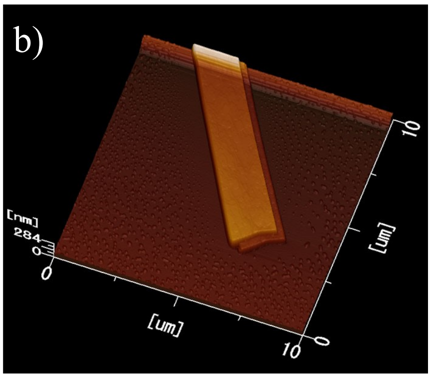

Figure S2. The AFM images of C_{70}/3,5-TPP nanoribbons.

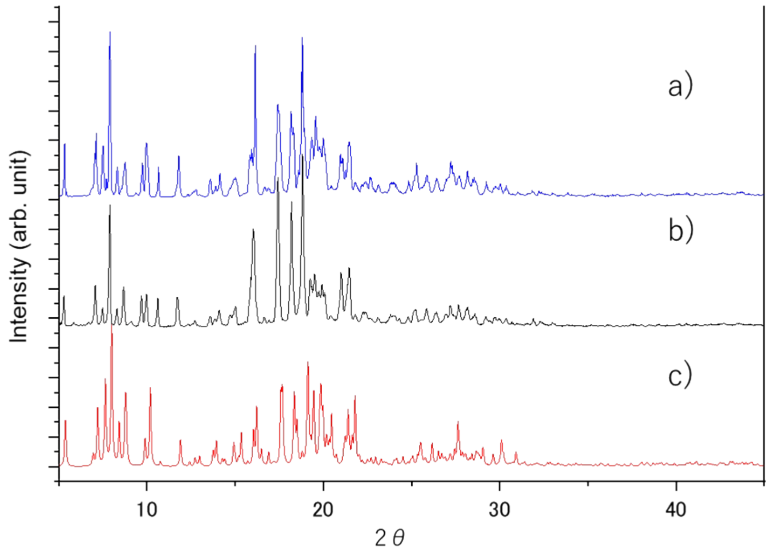

Figure S3. The experimental XRD pattern of C_{70}/3,5-TPP nanoribbons a)without annealing, b) after annealing at 80°C for 1h, and c) simulated XRD pattern from single crystal data

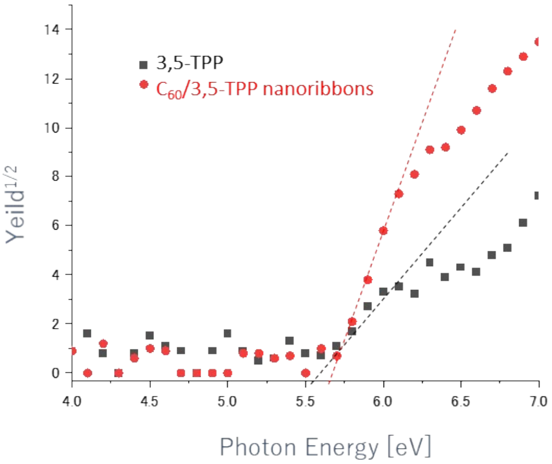

Figure S4. PYSA spectra of 3,5-TPP and C_{60}/3,5-TPP cocrystals.

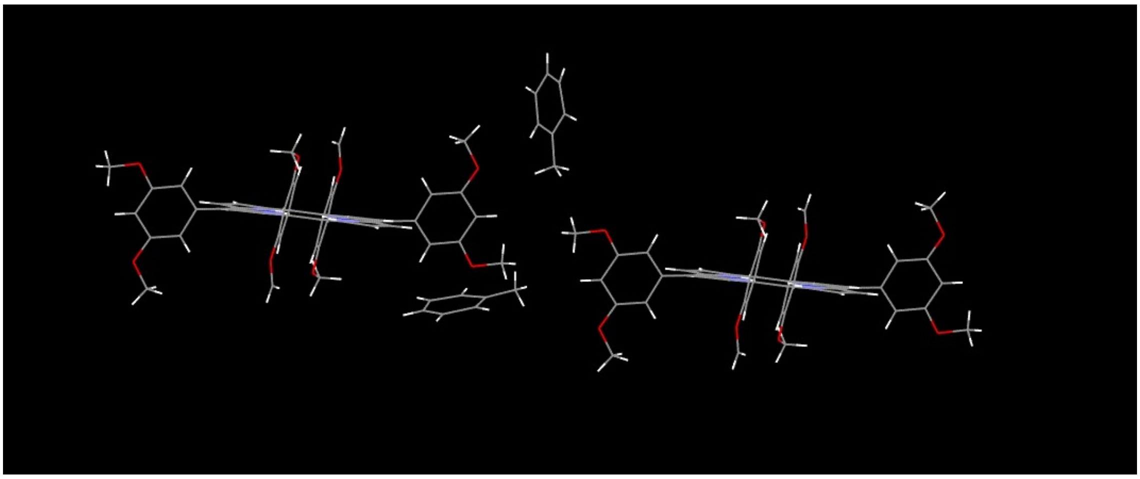

Figure S5. The [(3,5-TPP) toluene$_2$ (3,5-TPP)] unit structure in C_{60}/3,5-TPP cocrystals

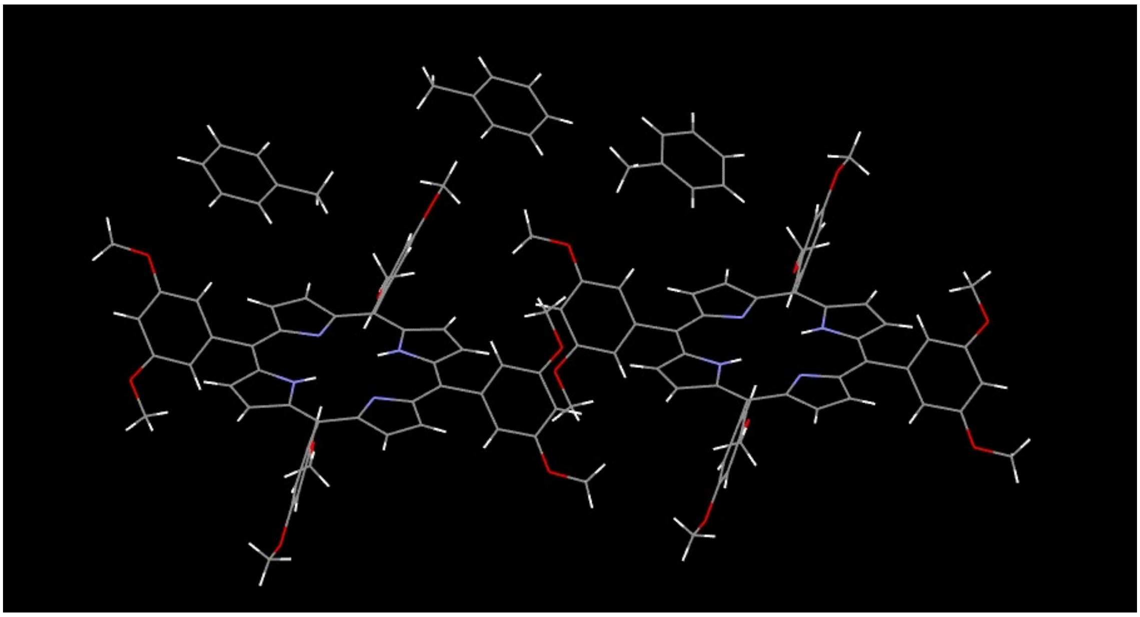

Figure S6. The [(3,5-TPP)$_2$ toluene$_3$] unit structure in C_{70}/3,5-TPP nanoribbons

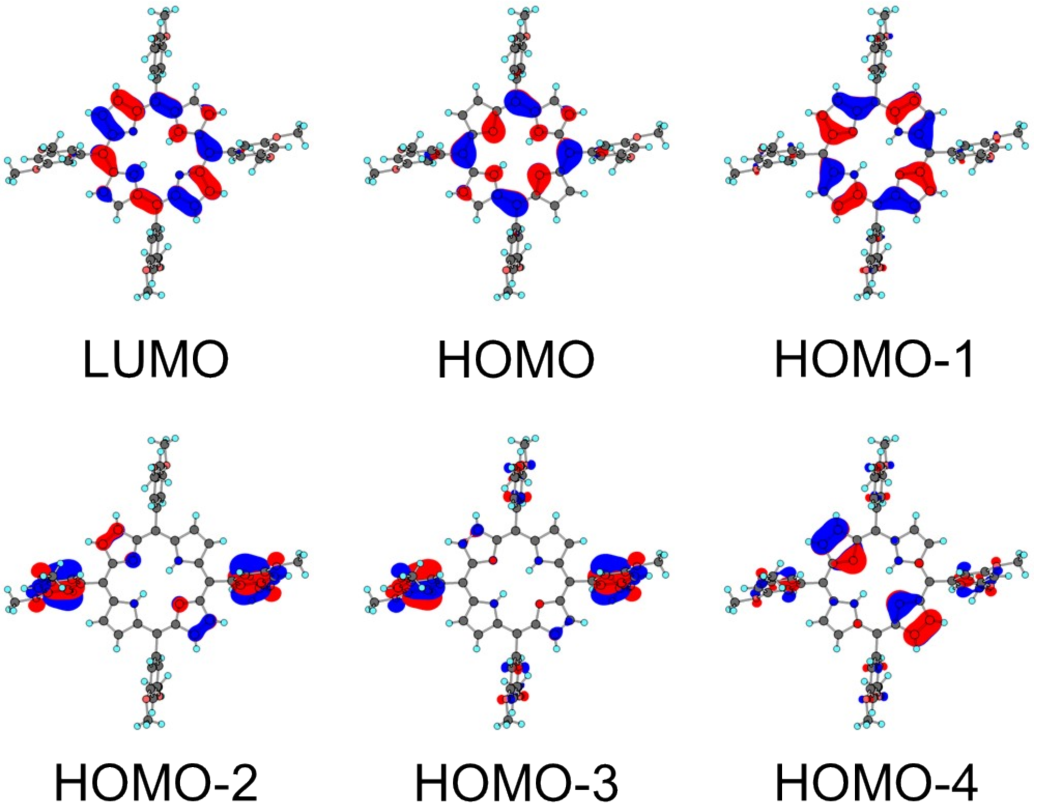

Figure S7. Molecular orbitals of 3,5-TPP.

Table S1. Coupling constant between 3,5-TPPs of C_{60}- and C_{70}-based cocrystals.

		**Coupling constant (eV)**	
**A**	**B**	**TPPs of C_{60} cocrystal**	**TPPs of C_{70} cocrystal**
HOMO	HOMO	1.47×10^{-6}	8.8×10^{-4}
HOMO-1	HOMO-1	9.2×10^{-5}	3.3×10^{-3}
HOMO-2	HOMO-2	2.3×10^{-5}	1.5×10^{-2}
HOMO-3	HOMO-3	4.1×10^{-4}	5.3×10^{-2}
HOMO-4	HOMO-4	7.7×10^{-5}	1.7×10^{-2}

Here, we explain the calculation technique for coupling constants. The coupling constants were evaluated based on $\langle \phi_A | H_{AB} | \phi_B \rangle$. Here, $\phi_{A(B)}$ is the molecular orbital for each TPP molecule $A(B)$, and H_{AB} is the Fock operator with both TPPs, which is obtained from the DFT calculation. This method assumes that a hole is localized on each TPP site, and hole-hopping occurs between those. To numerically estimate the coupling constants, we need to execute matrix operations between the Fock matrix and the coefficient vectors of molecular orbitals. For such matrix calculations based on quantum chemistry, we employed a Python-based quantum chemistry program tool.[1, 2] In the tool, the core part is developed by C++, but the Python interfaces are prepared. Thus, we can easily handle with matrix operations based on quantum chemistry without compromising calculation speeds. The tool has been employed to investigate the ground and excited electronic structures of various materials and molecules.[3-5]

1. T. Shimazaki, M. Hashimoto and T. Maeda, *Proceedings of the 3rd International Workshop on Software Engineering for High Performance Computing in Computational Science and Engineering*, 2015, 9 [DOI: 10.1145/2830168.2830170].
2. T. Shimazaki, in *Recent Advances of the Fragment Molecular Orbital Method*, eds. Y. Mochizuki, S. Tanaka and K. Fukuzawa, Springer, Singapore2021, pp. 589-600.
3. T. Shimazaki and T. Nakajima, *Physical Chemistry Chemical Physics*, 2016, **18**, 27554-27563.
4. T. Shimazaki, K. Kitaura, D. G. Fedorov and T. Nakajima, *J Chem Phys*, 2017, **146**, 084109.
5. T. Shimazaki and M. Tachikawa, *Physical Chemistry Chemical Physics*, 2021, **23**, 21078-21086.

We employed molecular geometries from the corresponding experimental ones. We show those

below in the XYZ-format.

220

TPPs from C60/3,5-TPP

C	8.909562000	9.029039000	20.865231000
C	9.222536000	8.570314000	22.163545000
H	8.805132000	7.844836000	22.611445000
C	10.238668000	9.353502000	22.669700000
H	10.654499000	9.264957000	23.520246000
C	10.542577000	10.320180000	21.670562000
C	11.507135000	11.340562000	21.749590000
C	12.415303000	11.353988000	22.923718000
C	13.431073000	10.382835000	22.998982000
H	13.468638000	9.687476000	22.352986000
C	14.373594000	10.434302000	24.007528000
C	14.315775000	11.436782000	25.006666000
H	14.915483000	11.441705000	25.742509000
C	13.349247000	12.401223000	24.858018000
C	12.391580000	12.340805000	23.896513000
H	11.694118000	12.986712000	23.889833000
C	15.702335000	8.720239000	23.128814000
H	15.810540000	9.230027000	22.299624000
H	14.987316000	8.059363000	23.015804000
H	16.541439000	8.259299000	23.341022000
C	14.258107000	13.515587000	26.797963000
H	14.175880000	12.784895000	27.444994000
H	14.183016000	14.374812000	27.262685000
H	15.128934000	13.460630000	26.351305000
C	11.702587000	12.316191000	20.759860000
C	12.662663000	13.392515000	20.882165000
H	13.240849000	13.562623000	21.617124000
C	12.568500000	14.092909000	19.755078000
H	13.066101000	14.871778000	19.534835000
C	11.563308000	13.448457000	18.919641000
C	11.214771000	13.900468000	17.611918000
C	11.949104000	15.137905000	17.160330000

C	11.347525000	16.388768000	17.293925000
H	10.464822000	16.469146000	17.638562000
C	12.054111000	17.498658000	16.919484000
C	13.333957000	17.420339000	16.418974000
H	13.801200000	18.205406000	16.158502000
C	13.913500000	16.198566000	16.307959000
C	13.244060000	15.028259000	16.672992000
H	13.665091000	14.181096000	16.589825000
C	10.424228000	18.984477000	17.794435000
H	10.647493000	18.815107000	18.733398000
H	9.698533000	18.386144000	17.518270000
H	10.139325000	19.915173000	17.688067000
C	15.865890000	17.051122000	15.212859000
H	16.069324000	17.736433000	15.883843000
H	15.292078000	17.435264000	14.517734000
H	16.699930000	16.732319000	14.809083000
O	13.197427000	13.405941000	25.804470000
O	11.475342000	18.762947000	17.051197000
O	15.377874000	9.575033000	24.156176000
O	15.211691000	15.992699000	15.818739000
N	11.042698000	12.401223000	19.576324000
H	10.397615000	11.872280000	19.295098000
N	9.709237000	10.112076000	20.607449000
C	10.294265000	13.347761000	16.767073000
C	9.981290000	13.806486000	15.468758000
H	10.398695000	14.531964000	15.020859000
C	8.965158000	13.023298000	14.962604000
H	8.549328000	13.111843000	14.112057000
C	8.661250000	12.056620000	15.961742000
C	7.696692000	11.036238000	15.882714000
C	6.788524000	11.022812000	14.708586000
C	5.772753000	11.993965000	14.633321000
H	5.735189000	12.689324000	15.279317000
C	4.830232000	11.942498000	13.624775000
C	4.888052000	10.940018000	12.625638000
H	4.288344000	10.935095000	11.889795000

C	5.854580000	9.975577000	12.774285000
C	6.812246000	10.035995000	13.735791000
H	7.509708000	9.390088000	13.742470000
C	3.501491000	13.656561000	14.503490000
H	3.393287000	13.146773000	15.332680000
H	4.216510000	14.317437000	14.616500000
H	2.662388000	14.117501000	14.291281000
C	4.945720000	8.861213000	10.834340000
H	5.027947000	9.591905000	10.187309000
H	5.020810000	8.001988000	10.369619000
H	4.074893000	8.916170000	11.280998000
C	7.501239000	10.060609000	16.872443000
C	6.541163000	8.984285000	16.750138000
H	5.962978000	8.814177000	16.015179000
C	6.635327000	8.283891000	17.877226000
H	6.137725000	7.505022000	18.097469000
C	7.640519000	8.928343000	18.712663000
C	7.989055000	8.476332000	20.020385000
C	7.254723000	7.238895000	20.471973000
C	7.856301000	5.988032000	20.338378000
H	8.739005000	5.907654000	19.993742000
C	7.149716000	4.878142000	20.712820000
C	5.869870000	4.956461000	21.213329000
H	5.402627000	4.171394000	21.473801000
C	5.290327000	6.178234000	21.324345000
C	5.959766000	7.348541000	20.959311000
H	5.538735000	8.195704000	21.042479000
C	8.779598000	3.392323000	19.837869000
H	8.556334000	3.561693000	18.898905000
H	9.505293000	3.990656000	20.114033000
H	9.064502000	2.461627000	19.944236000
C	3.337937000	5.325678000	22.419445000
H	3.134503000	4.640367000	21.748461000
H	3.911749000	4.941536000	23.114570000
H	2.503897000	5.644481000	22.823221000
O	6.006400000	8.970859000	11.827833000

O	7.728485000	3.613853000	20.581107000
O	3.825952000	12.801767000	13.476128000
O	3.992136000	6.384101000	21.813565000
N	8.161129000	9.975577000	18.055979000
H	8.806212000	10.504520000	18.337205000
N	9.494590000	12.264724000	17.024854000
C	18.511475360	9.029039000	39.681382720
C	18.824449360	8.570314000	40.979696720
H	18.407045360	7.844836000	41.427596720
C	19.840581360	9.353502000	41.485851720
H	20.256412360	9.264957000	42.336397720
C	20.144490360	10.320180000	40.486713720
C	21.109048360	11.340562000	40.565741720
C	22.017216360	11.353988000	41.739869720
C	23.032986360	10.382835000	41.815133720
H	23.070551360	9.687476000	41.169137720
C	23.975507360	10.434302000	42.823679720
C	23.917688360	11.436782000	43.822817720
H	24.517396360	11.441705000	44.558660720
C	22.951160360	12.401223000	43.674169720
C	21.993493360	12.340805000	42.712664720
H	21.296031360	12.986712000	42.705984720
C	25.304248360	8.720239000	41.944965720
H	25.412453360	9.230027000	41.115775720
H	24.589229360	8.059363000	41.831955720
H	26.143352360	8.259299000	42.157173720
C	23.860020360	13.515587000	45.614114720
H	23.777793360	12.784895000	46.261145720
H	23.784929360	14.374812000	46.078836720
H	24.730847360	13.460630000	45.167456720
C	21.304500360	12.316191000	39.576011720
C	22.264576360	13.392515000	39.698316720
H	22.842762360	13.562623000	40.433275720
C	22.170413360	14.092909000	38.571229720
H	22.668014360	14.871778000	38.350986720
C	21.165221360	13.448457000	37.735792720

C	20.816684360	13.900468000	36.428069720
C	21.551017360	15.137905000	35.976481720
C	20.949438360	16.388768000	36.110076720
H	20.066735360	16.469146000	36.454713720
C	21.656024360	17.498658000	35.735635720
C	22.935870360	17.420339000	35.235125720
H	23.403113360	18.205406000	34.974653720
C	23.515413360	16.198566000	35.124110720
C	22.845973360	15.028259000	35.489143720
H	23.267004360	14.181096000	35.405976720
C	20.026141360	18.984477000	36.610586720
H	20.249406360	18.815107000	37.549549720
H	19.300446360	18.386144000	36.334421720
H	19.741238360	19.915173000	36.504218720
C	25.467803360	17.051122000	34.029010720
H	25.671237360	17.736433000	34.699994720
H	24.893991360	17.435264000	33.333885720
H	26.301843360	16.732319000	33.625234720
O	22.799340360	13.405941000	44.620621720
O	21.077255360	18.762947000	35.867348720
O	24.979787360	9.575033000	42.972327720
O	24.813604360	15.992699000	34.634890720
N	20.644611360	12.401223000	38.392475720
H	19.999528360	11.872280000	38.111249720
N	19.311150360	10.112076000	39.423600720
C	19.896178360	13.347761000	35.583224720
C	19.583203360	13.806486000	34.284909720
H	20.000608360	14.531964000	33.837010720
C	18.567071360	13.023298000	33.778755720
H	18.151241360	13.111843000	32.928208720
C	18.263163360	12.056620000	34.777893720
C	17.298605360	11.036238000	34.698865720
C	16.390437360	11.022812000	33.524737720
C	15.374666360	11.993965000	33.449472720
H	15.337102360	12.689324000	34.095468720
C	14.432145360	11.942498000	32.440926720

C	14.489965360	10.940018000	31.441789720
H	13.890257360	10.935095000	30.705946720
C	15.456493360	9.975577000	31.590436720
C	16.414159360	10.035995000	32.551942720
H	17.111621360	9.390088000	32.558621720
C	13.103404360	13.656561000	33.319641720
H	12.995200360	13.146773000	34.148831720
H	13.818423360	14.317437000	33.432651720
H	12.264301360	14.117501000	33.107432720
C	14.547633360	8.861213000	29.650491720
H	14.629860360	9.591905000	29.003460720
H	14.622723360	8.001988000	29.185770720
H	13.676806360	8.916170000	30.097149720
C	17.103152360	10.060609000	35.688594720
C	16.143076360	8.984285000	35.566289720
H	15.564891360	8.814177000	34.831330720
C	16.237240360	8.283891000	36.693377720
H	15.739638360	7.505022000	36.913620720
C	17.242432360	8.928343000	37.528814720
C	17.590968360	8.476332000	38.836536720
C	16.856636360	7.238895000	39.288124720
C	17.458214360	5.988032000	39.154529720
H	18.340918360	5.907654000	38.809893720
C	16.751629360	4.878142000	39.528971720
C	15.471783360	4.956461000	40.029480720
H	15.004540360	4.171394000	40.289952720
C	14.892240360	6.178234000	40.140496720
C	15.561679360	7.348541000	39.775462720
H	15.140648360	8.195704000	39.858630720
C	18.381511360	3.392323000	38.654020720
H	18.158247360	3.561693000	37.715056720
H	19.107206360	3.990656000	38.930184720
H	18.666415360	2.461627000	38.760387720
C	12.939850360	5.325678000	41.235596720
H	12.736416360	4.640367000	40.564612720
H	13.513662360	4.941536000	41.930721720

H	12.105810360	5.644481000	41.639372720
O	15.608313360	8.970859000	30.643984720
O	17.330398360	3.613853000	39.397258720
O	13.427865360	12.801767000	32.292279720
O	13.594049360	6.384101000	40.629716720
N	17.763042360	9.975577000	36.872130720
H	18.408125360	10.504520000	37.153356720
N	19.096503360	12.264724000	35.841005720

220

TPPs from C70/3,5-TPP

O	7.036909000	5.578862000	27.054926000
O	8.730964000	9.940323000	26.223271000
O	17.332155000	4.226595000	24.945142000
O	15.420140000	-0.079525000	24.440080000
O	13.366989000	0.628907000	13.838282000
O	14.892249000	5.065670000	13.065816000
O	4.694636000	5.930698000	15.281360000
O	5.970166000	10.383322000	16.319126000
N	11.423852000	5.056033000	22.051776000
N	12.756550000	4.089217000	19.668881000
H	11.967534000	4.476511000	19.832928000
N	9.316905000	6.337768000	20.506545000
H	10.075813000	5.942146000	20.337690000
N	10.636535000	5.354012000	18.120344000
C	8.599522000	8.660258000	25.777098000
C	13.029807000	2.315469000	15.563185000
H	12.599953000	1.703747000	16.149370000
C	6.231854000	9.055661000	16.471456000
C	9.545608000	5.946145000	17.541070000
C	13.496289000	1.894704000	14.321411000
C	9.103875000	8.190515000	24.568073000
H	9.577145000	8.772135000	23.985194000
C	12.503477000	4.442696000	22.633755000
C	13.532070000	3.750877000	21.977264000
C	13.638247000	3.593229000	20.592174000

C	7.715383000	6.488314000	26.287869000
C	8.901859000	6.846362000	24.227359000
C	8.856073000	6.642648000	21.758534000
C	11.397435000	4.853730000	17.096098000
C	13.219009000	3.782099000	18.417492000
C	14.294858000	4.107044000	13.839484000
C	7.288568000	7.353927000	20.300434000
H	6.505340000	7.726601000	19.914051000
C	10.690289000	5.601092000	23.073919000
C	14.456052000	3.057581000	18.552095000
H	14.994153000	2.725531000	17.843927000
C	14.131293000	2.787326000	13.449495000
H	14.444964000	2.492029000	12.604020000
C	12.596405000	4.130506000	17.216880000
C	5.509889000	6.839832000	15.890078000
C	16.412807000	3.377829000	24.398618000
C	7.905147000	7.809943000	26.647211000
H	7.568348000	8.137079000	27.473458000
C	8.208791000	5.994422000	25.071633000
H	8.068855000	5.086396000	24.829468000
C	9.501511000	6.334023000	22.957042000
C	12.446612000	4.601797000	24.066617000
H	13.073392000	4.269333000	24.697268000
C	13.841170000	4.548550000	15.089971000
H	13.968843000	5.452215000	15.356173000
C	15.534363000	3.942762000	23.463907000
H	15.591882000	4.867878000	23.252088000
C	9.519074000	10.839208000	25.447200000
H	10.431979000	10.489267000	25.370284000
H	9.127150000	10.930169000	24.553051000
H	9.541157000	11.716310000	25.884059000
C	10.765401000	5.143120000	15.827284000
H	11.088232000	4.906151000	14.965583000
C	11.325608000	5.318388000	24.340630000
H	11.018383000	5.582755000	25.199026000
C	12.596459000	-0.299647000	14.598730000

H	11.688431000	0.049038000	14.716207000
H	12.559929000	-1.157485000	14.127318000
H	13.012659000	-0.428057000	15.476354000
C	3.661880000	6.443379000	14.444597000
H	3.078432000	7.029575000	14.968588000
H	4.061173000	6.953270000	13.709688000
H	3.136784000	5.699321000	14.082250000
C	18.200767000	3.692890000	25.944450000
H	17.669536000	3.378336000	26.704297000
H	18.708953000	2.944325000	25.568583000
H	18.819393000	4.389485000	26.244603000
C	15.284781000	4.696466000	11.749230000
H	15.924832000	3.955749000	11.795800000
H	14.496279000	4.415247000	11.239961000
H	15.704319000	5.463850000	11.306061000
C	14.596674000	-0.985227000	23.712983000
H	13.654184000	-0.759781000	23.859003000
H	14.803699000	-0.920315000	22.756340000
H	14.764236000	-1.900905000	24.021248000
C	14.581143000	3.141814000	22.852184000
C	8.390129000	6.760046000	19.590162000
C	7.242063000	8.538698000	17.273366000
H	7.825209000	9.117872000	17.747782000
C	13.203229000	3.653470000	15.936048000
C	7.386138000	7.147186000	17.368910000
C	7.576183000	7.285627000	21.634146000
H	7.031978000	7.603107000	22.344718000
C	5.368611000	8.212595000	15.760883000
H	4.695436000	8.577301000	15.196932000
C	8.490370000	6.590065000	18.204471000
C	9.621335000	5.819072000	16.105503000
H	8.990790000	6.147503000	15.476354000
C	6.522503000	6.297994000	16.690787000
H	6.618000000	5.356605000	16.768304000
C	14.709873000	2.939118000	19.890615000
H	15.456533000	2.504990000	20.283608000

C	16.327173000	2.034481000	24.722206000
H	16.899237000	1.662534000	25.382302000
C	14.518148000	1.767220000	23.125296000
H	13.890661000	1.212437000	22.675217000
C	15.389922000	1.231926000	24.065715000
C	6.821054000	11.307101000	16.976518000
H	6.776379000	11.160469000	17.946081000
H	7.742028000	11.178345000	16.669154000
H	6.533409000	12.221760000	16.771308000
C	6.622698000	5.998741000	28.351082000
H	7.406715000	6.272940000	28.870566000
H	6.005154000	6.755312000	28.266655000
H	6.171318000	5.257641000	28.804466000
O	-0.870124850	13.523736030	36.958170730
O	0.687456150	17.912600030	36.025262730
O	-3.076564850	13.766035030	24.913895730
O	-1.455304850	18.149646030	25.653911730
N	1.364177150	14.177812030	30.358110730
H	2.144755150	13.788424030	30.222605730
N	2.719192150	13.074677030	28.075866730
C	0.557756150	16.625307030	35.589304730
C	1.042847150	16.155638030	34.373970730
H	1.480925150	16.742737030	33.767955730
C	-0.099135850	15.774691030	36.484655730
H	-0.430283850	16.106145030	37.310301730
C	1.639694150	13.628377030	27.435299730
C	-0.258570850	14.441937030	36.148147730
C	0.475080150	14.594870030	29.405673730
C	0.884755150	14.536151030	31.588166730
C	0.594059150	14.352379030	28.031098730
C	-0.375814850	15.209262030	31.410598730
H	-0.931485850	15.558604030	32.097435730
C	3.488206150	12.484655030	27.106303730
C	1.503861150	14.265161030	32.810110730
C	0.219873150	13.942449030	34.929809730
H	0.098912150	13.027379030	34.702366730

C	-0.622193850	15.250133030	30.065468730
H	-1.378690850	15.640718030	29.645735730
C	-2.226835850	14.645559030	25.522012730
C	-1.344960850	16.830492030	25.968486730
C	1.725933150	13.370826030	26.016859730
H	1.103202150	13.641218030	25.352256730
C	-1.333473850	14.091838030	26.451615730
H	-1.335998850	13.156556030	26.623174730
C	1.495979150	18.799260030	35.257002730
H	2.396877150	18.423448030	35.165064730
H	1.548422150	19.667678030	35.708884730
H	1.099686150	18.916138030	34.368862730
C	-1.325044850	13.975007030	38.227585730
H	-1.973653850	14.699401030	38.103498730
H	-0.563964850	14.303952030	38.749473730
H	-1.752098850	13.232202030	38.704405730
C	-4.066555850	14.318356030	24.049490730
H	-3.629505850	14.762982030	23.294150730
H	-4.605888850	14.969638030	24.544036730
H	-4.646292850	13.600643030	23.714785730
C	-0.658603850	19.068624030	26.392426730
H	0.289970150	18.891509030	26.220566730
H	-0.842612850	18.964892030	27.350271730
H	-0.874019850	19.983484030	26.115408730
C	0.872697150	14.800361030	34.058494730
C	-0.444538850	14.923099030	27.119823730
C	-2.220880850	16.004287030	25.260317730
H	-2.804938850	16.369758030	24.607132730
C	-0.457668850	16.307943030	26.899891730
H	0.130138150	16.879731030	27.380316730
C	2.868520150	12.666731030	25.814654730
H	3.197794150	12.353616030	24.979694730
O	7.062825150	12.523541030	23.132506730
O	5.505244150	8.134677030	24.065414730
O	9.269265150	12.281242030	35.176781730
O	7.648005150	7.897631030	34.436765730

N	4.828523150	11.869465030	29.732566730
H	4.047945150	12.258853030	29.868071730
N	3.473508150	12.972600030	32.014810730
C	5.634944150	9.421970030	24.501372730
C	5.149853150	9.891639030	25.716706730
H	4.711774150	9.304540030	26.322721730
C	6.291836150	10.272586030	23.606021730
H	6.622984150	9.941132030	22.780375730
C	4.553006150	12.418900030	32.655377730
C	6.451271150	11.605340030	23.942529730
C	5.717620150	11.452407030	30.685003730
C	5.307944150	11.511126030	28.502510730
C	5.598641150	11.694898030	32.059578730
C	6.568515150	10.838015030	28.680078730
H	7.124185150	10.488673030	27.993241730
C	2.704494150	13.562622030	32.984373730
C	4.688839150	11.782116030	27.280566730
C	5.972827150	12.104828030	25.160867730
H	6.093788150	13.019898030	25.388310730
C	6.814894150	10.797144030	30.025208730
H	7.571391150	10.406559030	30.444941730
C	8.419536150	11.401718030	34.568664730
C	7.537661150	9.216785030	34.122190730
C	4.466767150	12.676451030	34.073817730
H	5.089497150	12.406059030	34.738420730
C	7.526174150	11.955439030	33.639061730
H	7.528699150	12.890721030	33.467502730
C	4.696721150	7.248017030	24.833674730
H	3.795822150	7.623829030	24.925612730
H	4.644278150	6.379599030	24.381792730
H	5.093014150	7.131139030	25.721814730
C	7.517745150	12.072270030	21.863091730
H	8.166354150	11.347876030	21.987178730
H	6.756665150	11.743325030	21.341203730
H	7.944799150	12.815075030	21.386271730
C	10.259256150	11.728921030	36.041186730

H	9.822206150	11.284295030	36.796526730
H	10.798589150	11.077639030	35.546640730
H	10.838993150	12.446634030	36.375891730
C	6.851304150	6.978653030	33.698250730
H	5.902729150	7.155768030	33.870110730
H	7.035313150	7.082385030	32.740405730
H	7.066720150	6.063793030	33.975268730
C	5.320003150	11.246916030	26.032182730
C	6.637239150	11.124178030	32.970853730
C	8.413581150	10.042990030	34.830359730
H	8.997639150	9.677519030	35.483544730
C	6.650369150	9.739334030	33.190785730
H	6.062561150	9.167546030	32.710360730
C	3.324180150	13.380546030	34.276022730
H	2.994906150	13.693661030	35.110982730
